# Doxorubicin-triggered self-assembly of native amphiphilic peptides into spherical nanoparticles

**DOI:** 10.18632/oncotarget.11213

**Published:** 2016-08-11

**Authors:** Xiaobo Fan, Fengfeng Zhao, Xiyong Wang, Guoqiu Wu

**Affiliations:** ^1^ Center of Clinical Laboratory Medicine of Zhongda Hospital, Southeast University, Nanjing, 210009, China; ^2^ Medical School, Southeast University, Nanjing, 210009, China

**Keywords:** matrilin-1 C-terminal peptide, self-assembly, nanosphere, drug delivery, cancer therapy

## Abstract

In this study, we designed and fabricated self-assembly nanospheres, which consisted of a P45 peptide and doxorubicin (Dox). P45 is a hybrid peptide composed of an Arg-Gly-Asp motif linked to the human matrilin-1 C-terminal domain by a serine linker. The fabricated nanospheres had a uniform mulberry-like spherical shape, a diameter of 63 nm, excellent polydispersity, and high Dox drug-loading efficiency. In the presence of the RGD motif, the Dox/P45 nanospheres could specifically target A549 cells, which have high integrin α_v_β_3_ expression. Confocal laser scanning microscopy and flow cytometry results showed the enhanced cellular uptake of Dox/P45, and the CCK8 assay indicated the low cytotoxicity of the nanospheres to normal human embryonic kidney 293 cells. Furthermore, the fabricated nanospheres were stable in a physiological environment, but they disassembled and exhibited a rapid Dox release in an acidic atmosphere, allowing for a specific pH-sensitive release into cytosol after cellular uptake. These results suggest that natural amphiphilic peptides can be used as carriers of nanodrugs for targeting delivery as well as controlled drug release for cancer therapy.

## INTRODUCTION

As a nanoscale drug delivery system for tumor therapy, nanosphere-based drug delivery has been extensively investigated over the past decades. As alternatives to classical nanosphere-based delivery systems, such as liposome and polymer vehicles, peptide-based nanoparticles represent a novel class of drug carriers [1]. Native amphiphilic peptides, particularly human-derived peptides, are considered powerful and promising tools in clinical anti-tumor research owing to their high biocompatibility, non-toxicity, non-immunogenicity, and diversiform structures. The self-assembling capabilities of peptide amphiphiles are attributed to their protein nature resulting from intra- or intermolecular hydrogen bonds and amphiphilic and electrostatic interactions, leading to well-defined nanostructures, such as nanofibers, nanotubes, and ribbons [2–4]. However, uniform spherical micelles formed by self-assembly native peptides have been rarely reported.

Previously reported as “cartilage matrix protein” (CMP) [5, 6], matrilin-1 is the prototype member of the human matrilin family; it is an adaptor in the assembly of the extracellular matrix structure. The last 28 amino acids at the C-terminus of matrilin-1 (Q466-T494) are known to form a coiled coil of four α-helices and have the capability to self-assemble into a stable (*T*_m_>60°C) homodimer or trimer [7–11]. The coiled-coli motif has multiple functions [12–16]. Most recent studies have shown that it is a potent carrier for cancer treatment owing to its structural stability and specificity [16–18].

**Figure 1:**
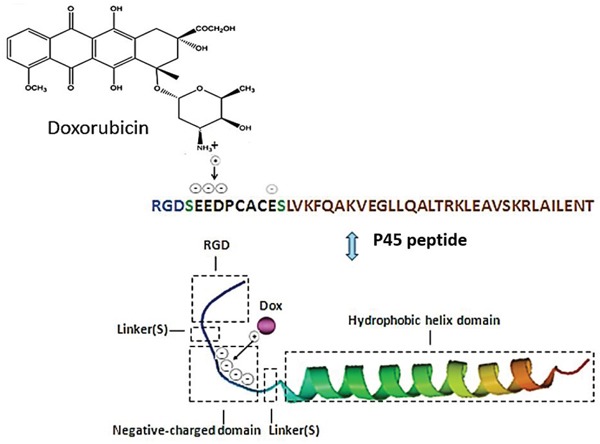
Schematic representation of the P45 peptide structure and P45 combined with Doxorubicin (Dox)

**Figure 2:**
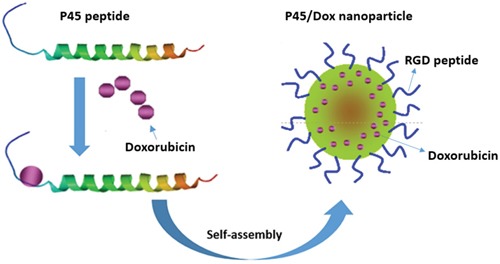
Schematic representation of Dox/P45 nanoparticles self-assembly triggered by Dox

In this study, a 41-residue peptide (P41) based on the C-terminal domain of human matrilin-1 is designed to generate a self-assembly nanosphere. The Arg-Gly-Asp (RGD) peptide is considered an excellent ligand for integrin α_v_β_3_, which is overexpressed in various tumor cells, and thus shows good targeting potential [19–22]. Therefore, we synthesize a so-called P45 biofunctional hybrid (hereafter referred to as P45), which consists of the RGD peptide linked to the N-terminal domain of P41 with a serine linker (Figure 1). In the presence of a cationic drug, such as doxorubicin (Dox), P45 self-assembles into a spherical nanoparticle (Dox/P45) (Figure 2). Particle size, polydispersity index (PDI), and microscopic morphology are obtained by DLS and TEM. The cellular uptake of Dox/P45 is also investigated by flow cytometry (FCM) and confocal laser scanning microscopy (CLSM). In addition, the cytotoxicity of the Dox/P45 nanoparticles is confirmed by CCK-8 assay.

## RESULTS

### P45 peptide synthesis and critical micelle concentration determination

P45 peptide was synthesized, refolded, and purified as previously reported [9]. As shown in Supplementary Figure S1-S2, the P45 peptide of >95% purity was obtained, and the molecular weight by mass spectrometry matched the theoretical value.

**Figure 3:**
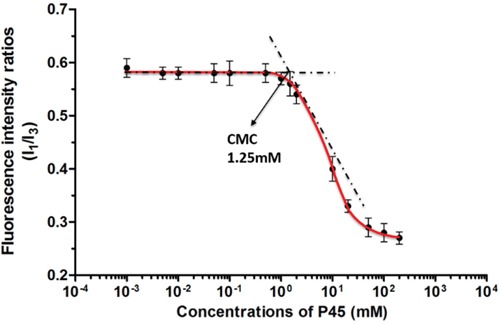
Fluorescence intensity ratios from pyrene excitation spectra are plotted against the concentrations of Dox/P45 nanoparticles

The critical micelle concentration (CMC) was determined by fluorescence spectroscopy using pyrene as the hydrophobic fluorescent probe. Pyrene is sensitive to environmental hydrophobicity. The CMC of P45 peptide in PBS was estimated by measuring the fluorescence intensity at wavelengths of 373 and 384 nm, which correspond to the first vibronic band (*I*_1_) and the third vibronic band (*I*_3_) of pyrene, respectively. The ratios of *I*_1_ to *I*_2_ are plotted in Figure 3, which shows that a sudden slip appeared at a concentration of 1.25 mM, which was assumed the CMC of the peptide.

### CD analysis of the peptide structure

**Figure 4:**
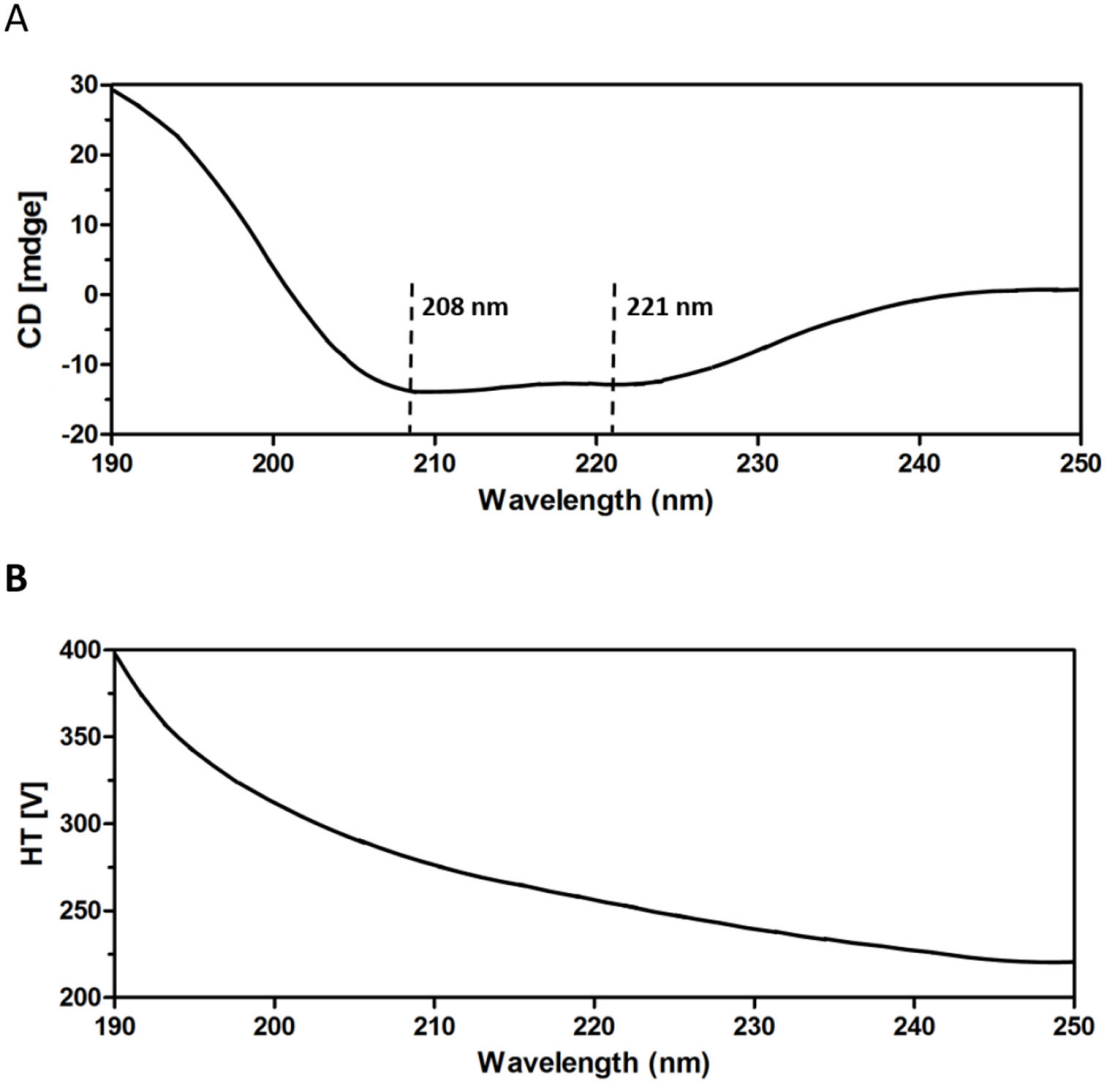
CD spectra of the P45 peptide at 0.5 mg/mL in 50 mM PBS (pH 7.4) **A.** Absorption at wavelengths between 190 and 250 nm. **B.** HT voltage is maintained below 600 V throughout the measurements.

CD spectroscopy was employed to characterize the secondary structure of P45 in the aqueous buffer. The result demonstrated that the peptide demonstrated maximum absorption levels at 208 and 221 nm; these peaks are usually considered typical of an α-helix (Figure 4).

### Characterization of self-assembled Dox/P45 nanoparticles

**Figure 5:**
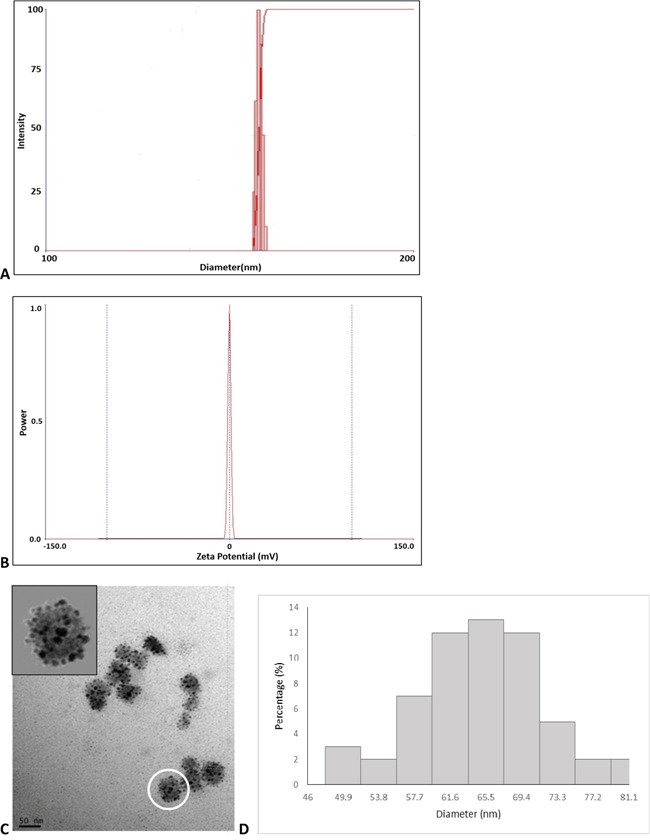
Size and morphology of the prepared nanospheres The shape and charge of the prepared nanospheres were characterized by **A.** average hydrodynamic diameter, **B.** zeta potential, **C.** transmission electron microscopy (TEM) image, and **D.** TEM size distribution.

P45 formed into nanoparticles via self-assembly in the presence of Dox; however, the average hydrodynamic diameter of the Dox/P45 nanoparticles and the corresponding PDI varied when the molar ratio of Dox to peptide shifted, as shown in Table 1. An obtained neutral and homogeneous Dox/P45 nanoparticle demonstrated a PDI of 0.189 and a corresponding diameter of 167 ± 3.3 nm when the molar ratio of Dox to peptide was set to 8 (Figure 5A and 5B). TEM was employed to observe the structure of the Dox/P45 nanoparticles, which exhibited a uniform spherical shape resembling a mulberry with a diameter of 63 nm (Figure 5C).

**Table 1:** Dox/P45 molar ratio on particle size and PDI (mean±SD; n = 3)

DOX/P45 molar ratio	16:1	10:1	8:1	4:1	2:1	1:1
Particle size(nm)	372±8.9	209±5.5	167±3.3	218±7.5	292±9.1	279±4.5
PDI	0.223	0.277	0.189	0.208	0.303	0.229

**Figure 6:**
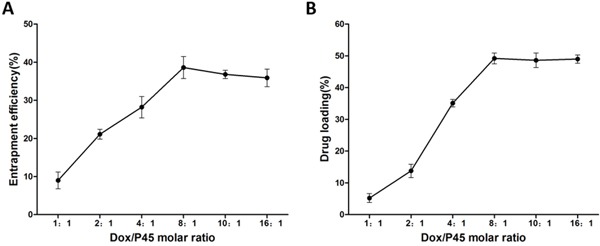
Effect of the Dox/P45 molar ratio on the **A.** entrapment efficiency and **B.** drug loading of the Dox/P45 nanoparticles.

Entrapment efficiency (EE) and drug loading (DL) at various Dox/P45 molar ratios are shown in Figure 6, revealing that the EE and DL values increased with the peptide proportion and reached a plateau at a molar ratio below 8:1 (Dox:peptide). The maximal EE and DL reached 38.6 and 49.2%, respectively.

### pH-Dependent drug release

**Figure 7:**
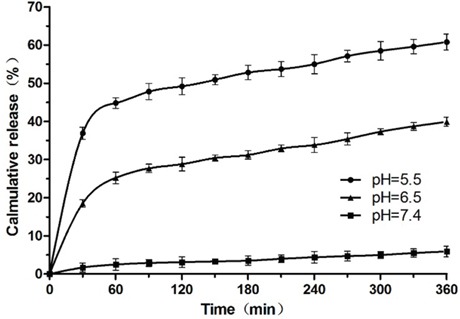
*In vitro* release of Dox from Dox/P45 nanoparticles at different pH levels Data are presented as mean ± SD (*n*=3).

The drug release rates of Dox/P45 nanoparticles into aqueous solutions at different pH and a temperature of 37°C were verified and compared. As shown in Figure 7, the Dox release was characterized by binary kinetics in acidic buffers: a rapid release in the first hour followed by a slow release lasting for several hours. The total release reached 60.8% and 39.9% after 6 h in pH 5.5 and pH 6.5 buffers, respectively. By contrast, the release at physiological pH 7.4 was negligible.

### FCM analysis

**Figure 8:**
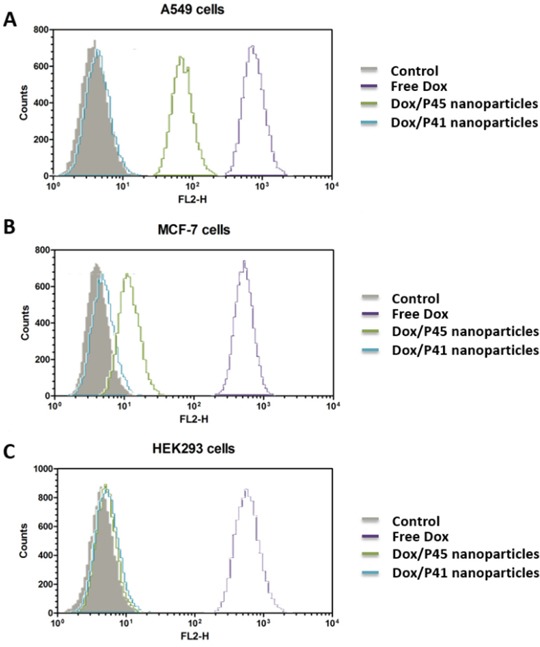
Flow cytometric measurement of Dox uptake by integrin α_v_β_3_ high-expressed A549 cells **A.** low-expressed MCF-7 cells **B.** and negative-expressed HEK293 cells **C.** after incubated with free Dox, Dox/P45 nanoparticles and Dox/P41 nanoparticles for 2.0 h. Untreated cells served as negative control.

The roles of the targeting RGD peptide during the cell internalization of Dox/P45 nanoparticles were investigated by FCM. As shown in Figure 8, the Dox/P45 nanoparticle group clearly showed stronger geometric mean intensity values in both A549 and MCF-7 cells than the Dox/P41 nanoparticles group. The free Dox group showed the strongest geometric mean intensity value among the three cell lines. However, both the absence and presence of the RGD peptide of the nanoparticles did not influence the cellular uptake by human embryonic kidney 293 (HEK293) cells.

### Confocal laser scanning microscopy

**Figure 9:**
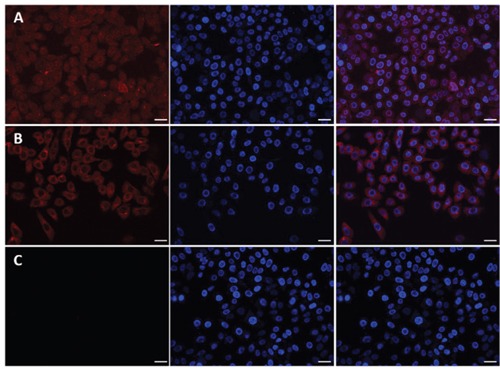
Laser scanning confocal microscopy (LSCM) images of A549 cells incubated with free DOX **A.** Dox/P45 nanoparticles **B.** or Dox/P41 nanoparticles **C.** at 37°C for 2.0 h. All images are obtained in the same scale bar (30μm).

**Figure 10:**
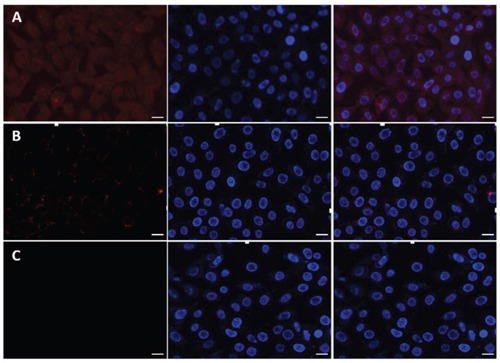
Laser scanning confocal microscopy (LSCM) images of MCF-7 cells incubated with free DOX **A.** Dox/P45 nanoparticles **B.** or Dox/P41 nanoparticles **C.** at 37°C for 2.0 h. All images are obtained in the same scale bar (20μm).

**Figure 11:**
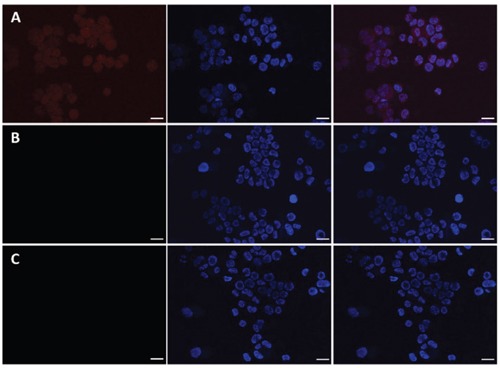
Laser scanning confocal microscopy (LSCM) images of HEK293 cells incubated withfree DOX **A.** Dox/P45 nanoparticles **B.** or Dox/P41 nanoparticles **C.** at 37°C for 2.0 h. All images are obtained in the same scale bar (30μm).

The targeting effect and cell penetrating efficiency of the Dox/P45 nanoparticles were further evaluated by CLSM. The cell nuclei were stained with Hoechst 33342 blue in contrast to the red fluorescent Dox in the nanocarrier to clarify the internalization of the nanocarrier. As shown in Figure 9, the strong red fluorescence presented in the A549 cellular cytoplasm reveal the specific targeting of Dox/P45 to A549 cells, whereas a significantly weaker fluorescence was found in MCF-7 cells (Figure 10) and almost no red fluorescence showed in HEK293 cells (Figure 11). Free Dox mainly presented in the nuclei of A549, MCF-7, and HEK293 cells, whereas the Dox/P41 nanoparticles exhibited no intense intracellular fluorescence in these cells as expected.

### *In vitro* cytotoxicity studies

**Figure 12:**
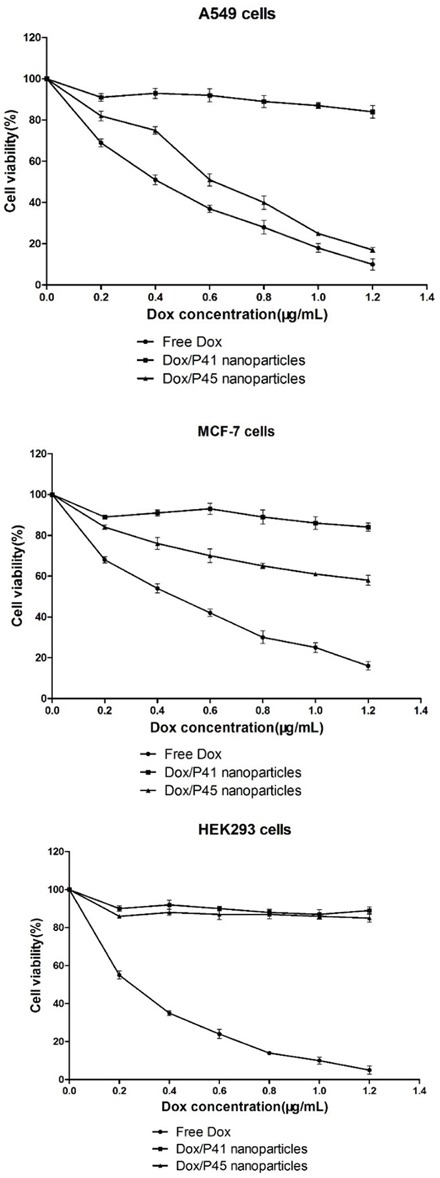
Cytotoxicity study using CCK8 assay A549, MCF-7, and HEK293 cells are treated with Dox, Dox/P41 nanoparticles, or Dox/P45 nanoparticles at desired concentrations. The CCK8 assay compares the cytotoxicity of the Dox/P45 nanoparticles with those of Dox and Dox/P41 nanoparticles.

The drug efficacy of Dox/P45 nanoparticles was investigated by CCK8 assay using A549, MCF-7, and HEK293 cells separately (Figure 12). The IC50 value of the Dox/P45 nanoparticles was 0.57 μg/mL for A549 cells; this value was comparable to that of free Dox (0.39 μg/mL). For MCF-7 cells, the IC50 value of the Dox/P45 nanoparticles was 2.2 μg/mL, whereas that of free Dox was 0.42 μg/mL. The IC50 value of the Dox/P45 nanoparticles for MCF-7 cells was approximately twice as high as that for A549 cells. By contrast, the Dox/P45 nanoparticles had no apparent cytotoxicity for HEK293 cells, whereas free Dox caused a strong inhibition, exhibiting an IC50 value of 0.24 μg/mL. The Dox/P41 nanoparticles also exhibited unspecific but lower cytotoxicity for A549, MCF-7, and HEK293 cells than free Dox did as expected.

## DISCUSSION

Amphiphilic peptides tend to self-assemble into high-aspect-ratio nanostructures, such as nanofibers, nanotubes, and ribbons, under certain conditions. Generally, they consist of two typical regions, the hydrophobic tail and the hydrophilic head. In aqueous solutions, the aggregation of hydrophobic tails drives self-assembly, leaving the hydrophilic head containing functional motifs present on the surface of the nanostructure [2, 3].

Matrilin-1 is a member from the matrilin protein family, of which the members share a basic skeleton composed of three segregated domains, namely, the von Willebrand factor A (vWFA) domain, the epidermal growth factor (EGF)-like domain, and the C-terminal α-helical coiled-coil module [7, 23]. The C-terminal coiled-coil domain has a distinctive heptad repeat, in which hydrophobic residues reside in the first and the forth positions whereas polar residues are usually present in the fifth and seventh sites [8]. The hydrophobic and ionic interactions within this α-helical coiled-coil are responsible for the formation of a parallel, disulfide-linked dimer or trimer with a length of approximately 5.1 nm and a diameter of 3 nm [8].

The protein sequence of the C-terminal module of matrilin-1 was analyzed, and a rich, negatively charged region (5 Glu, 6 Glu, 7 Asp, and 12 Glu in P45) was found at the N-terminus of the matrilin-1 coiled-coil domain (Figure 1). These acidic amino-acid residues provide intermolecular electrostatic repulsion; as a result, the peptide can be well dissolved in an aqueous buffer. Once the negative charges are neutralized or sterically blockaded by cationic drugs with hydrophobic aromatic rings, such as Dox, the force balance between intra- or/and intermolecular bonds will be broken. The coiled coils cross-link with one another, form the hydrophobic core via hydrophobic and aromatic interactions, and finally self-assemble into spherical nanoparticles (Figure 2). In the case of decreasing negative charges by adjusting pH to 6.0 or 4.0 or masking ionic interaction by dissolution in organic solvent, no nanospheres or defects were observed (data not shown). Moreover, the deletion of mutant P45 without the charge tail of eight amino-acid residues (EEDPCACE) could not form spherical particles even in the presence of Dox (data not shown). These results clearly implied that the negatively charge region was crucial in nanosphere self-assembly. Furthermore, the first two heptad repeats were more flexible than the other repeats, rendering flexibility to the functional motif, such as the RGD motif [10, 24] (Figure 1) at the N-terminus. Meanwhile, the P41 without the RGD motif demonstrated a TEM structure similar to that of the P45 peptide (Supplementary Figure S3), suggesting that the RGD peptide was unnecessary for the nanoparticle assembly and was probably present at the surface for targeting.

P45 consisted of an RGD motif followed by a 41-residue peptide derived from the C-terminus of matrilin-1. The CD spectra demonstrated two absorption peaks at 208 and 221 nm; these peaks are characteristic of an a-helix (Figure 4). By pyrene fluorescence assay, the CMC of the peptide was determined to be 1.25 mM (Figure 3).

TEM was also employed to investigate the nanoparticle shape, and the yielded nanoparticles showed a uniform spherical shape with a diameter of 63 nm (Figure 5), which was significantly smaller than the diameter indicated by DLS (Table 1). TEM images depicted the actual size in vacuum, whereas the size measured by the DLS method was the hydrodynamic diameter; the nanoparticles exhibited a larger hydrodynamic volume due to the effect of the solvent in the hydrated state. If used as drug nanocarriers, the Dox/P45 nanoparticles could benefit from their small size, which could facilitate tumor penetration, permit their accumulation in tumor tissues by the enhanced permeability retention effect, and bypass the elimination by the kidney [25, 26]. The mulberry-like nanoparticles in the TEM image suggested that at least two different substructures of different electron density were present. P45 consisted of elements similar to Dox, except for elemental sulfur. Elemental sulfur analysis by TEM was performed, and the result did not indicate a specific distribution of sulfur (data not shown). How the molecules assembled remains unknown, however.

For the evaluation of the kinetics of drug release, P45/Dox nanoparticles were incubated in buffers of different pH levels. No apparent release was observed after 6 h in physiological pH buffer. By contrast, the acidic buffer of pH 6.5 or 5.5 significantly increased Dox release (Figure 7). This result may be attributed to the protonation of acidic amino acids in the P45/Dox nanoparticles. At physiological pH, the rich, negatively charged domain remained deprotonated/deionized in the presence of Dox. By contrast, acidic pH allows for carboxyl group protonation, causing the structural transformation or disassembly between P45 peptide and Dox because of charge repulsion and consequently leading to the specific release of encapsulated Dox [27, 28]. These data suggested that the nanoparticles are stable at the blood pH, but the drug is released at an appreciable rate at the tumor interstitial pH (6.5) and late endosomal pH (5.5) [11, 12].

A549, MCF-7, and HEK293 cell lines, which respectively represent cell models with high, low, and negative expression of integrin α_v_β_3_, were used in this study [29–33] to evaluate the targeting effect during the cellular uptake of P45/Dox nanoparticles. After being exposed to Dox/P45 nanoparticles, A549 showed a significantly higher intracellular uptake than MCF-7 or HEK293 cells, according to the CLSM (Figures 9–11) and FCM results (Figure 8). Non-targeting Dox/P41 nanoparticles with shapes and sizes similar to DOX/P45 had weaker cellular internalization as a result of the absence of the RGD peptide. These results indicated the merits of the RGD peptide for targeting delivery in cancer cells expressing α_v_β_3_. The normal cell line showed no intracellular uptake after being co-incubated with both Dox/P45 and Dox/P41 nanoparticles, implying that the nanoparticles were considerably large to penetrate the cytomembrane freely and the intracellular uptake of the nanoparticles relied on a receptor-mediated endocytosis. As a small hydrophobic molecule, free Dox exhibited higher cellular uptake, and it was mainly present in the nuclei of the three cell lines (Figures 9–11), indicating its unspecific delivery and nucleophilic nature [34, 35].

The cytotoxicity results was consistent the CLSM and FCM results to a certain extent (Figures 8–11), indicating that the RGD peptide significantly and effectively enhanced the cellular uptake in α_v_β_3_-expressing cell lines. As expected, the Dox/P45 nanoparticles exhibited higher cytotoxicity than Dox/P41 in A549 and MCF-7 cells, depending on the α_v_β_3_ expression level. By contrast, the cytotoxicity levels of Dox/P45 and Dox/P41 are comparable in HEK293 cells, which were free of α_v_β_3_ expression. These results suggested the Dox/P45 nanoparticles could be smart and powerful carriers for cancer-targeting delivery.

## MATERIALS AND METHODS

### Materials

All Chemical Reagents utilized in this study were purchased from Sigma-Aldrich Company (St. Louis, MO, USA) unless otherwise mentioned. Doxorubicin hydrochloride (DOX·HCL) was purchased from the Zhejiang Hisun Pharmaceutical. Trypsin was obtained from Solarbio. Dulbecco's Modified Eagle's Medium (DMEM), Roswell Park Memorial Institute 1640 (RPMI-1640) and fetal bovine serum (FBS) were obtained from GIBCO, Invitrogen Corp. (Carlsbad, USA). Kit-8 (CCK-8) was purchased from DOJINDO Co. Ltd (DOJINDO, Japan).

### Cell lines

A549 cells and MCF-7 cells were purchased from Institute of Basic Medical Sciences, Chinese Academy of Medical Sciences (Beijing, China). A549 and MCF-7 cell lines were cultured in RPMI-1640 and DMEM medium, respectively, supplemented with 10% fetal bovine serum, 100 IU/mL penicillin and 100 IU/mL streptomycin. Human Embryonic Kidney 293 cells were provide by Cell Bank, Chinese Academy of Sciences (Shanghai, China) and grown in DMEM medium supplemented with 10% fetal bovine serum, 100 IU/mL penicillin and 100 IU/mL streptomycin. All cell lines were cultured in a humidified incubator (5% CO2/95% air atmosphere at 37°C).

### Synthesis of P45 peptide

P45 (RGDSEEDPCACESLVKFQAKVEGLLQALTRKLEAVSKRLAILENT) and the other peptides were synthesized by the solid-phase methodology with 9-fluorenyl-methoxy-carbonyl as protecting group. The crude compounds were purified by reverse-phase high-performance liquid chromatography (RP-HPLC) using an appropriate 0-60% acetonitrile gradient in 0.05% trifluoroacetic acid. Molecular weights were determined by electrospray mass spectrometry using an API instrument (Perkin Elmer SCIEX). The peptides were taken up in oxidation buffer (1 mg/1 ml) (100 mM ammonium acetate, pH 8.5) to refold for 3 days at RT under stirring and purified by RP-HPLC. The prepared peptides were lyophilized and stored in −20°C.

### Circular dichroism (CD) spectroscopy

CD measurements were performed with a Jasco-810 spectropo-larimeter (JASCO, Tokyo, Japan) using a 1-mm path-length quartz cell at 25°C. Each spectrum was obtained by averaging our scans in the 250–190 nm wave length range, with a 0.2 nm step resolution at 100 nm/min speeds. The peptide was dissolved in in 50 mM PBS (pH 7.4) at 100 nM (0.5 mg/ml). For a flexible peptide, the estimated percentages of secondary structure components should not be taken as absolute measures, but rather reflect on relative changes between spectra in a series of experiments.

### CMC measurement

The critical micelle concentration (CMC) of P45 was determined by the fluorescence method by using pyrene as the hydrophobic probe (reference). Briefly, a 1 μL aliquot of 2 mM pyrene was added to each vial containing acetone. After evaporating acetone overnight, a series of freshly prepared P45 concentrations in PBS ranging from 1×10^-3^ mM to 2×10^2^ mM were added to the vials and shaken vigorously for 24 h in darkness before sent for measurement. The pyrene fluorescence measurements were performed with a LS-55 Fluorescence Spectrometer (Perkin-Elmer, USA) at RT. The pyrene excitation wavelength was 335 nm and emission spectra were recorded from 360 to 440 nm at a scan speed of 300 nm/min using a 5 nm bandwidth. Five characteristic peaks of pyrene fluorescence spectra were observed for each sample. The ratio of the OD_373_ (I_1_) to OD_384_ (I_3_) was plotted as a function of P45 concentrations. A sudden fail of the OD_373_/OD_384_ value indicated the CMC where the pyrene shifted from water into the hydrophobic micellar core.

### Preparation and properties of Dox/P45 nanoparticles

P45 peptide dissolved in 0.1 M PBS (pH7.4) at 0.5 mg/mL was mixed with various amounts of DOX and the P45/DOX molar ratio ranged from 1 to 16. The mixed solution was sonicated under stirring for 10 min. To separate the uncombined doxorubicin from Dox/P45 nanoparticles, the dispersion was centrifuged at 20,000 rpm for 30 min. The supernatant containing free DOX and peptide was taken away for measurement by UV-Vis spectroscopy at 485 nm. Besides, Dox/P41 nanoparticles were also prepared in the same way.

Entrapment efficiency (EE) of doxorubicin was calculated using the following equations:

$$\text{EE}\left(\%\right)=\left[\left({\text{Dox}}_{\text{total}}-{\text{Dox}}_{\text{supernatant}}\right)/{\text{Dox}}_{\text{total}}\right]\times 100\%$$

Where, Dox_total_ and Dox_supernatant_ represent the absorbance of total Dox/P45 nanoparticles solution and the supernatant with free Dox, respectively.

Drug loading (DL) of DOX in P45 was calculated using the following equations:

Where, W _Dox_ and W _Dox/P45_ represent the total weight of DOX loaded in Dox/P45 nanoparticles and the weight of Dox/P45 nanoparticles, respectively.

Dox/P45 nanoparticles, which were precipitated as pellets, were washed twice, and then resuspended in 200 μL PBS for further experiments. Particle size, polydispersity index (PDI) and zeta potential of Dox/P45 nanoparticles were measured by dynamic light scattering (DLS) analysis using a Zetasizer Nano ZS system (Malvern Instruments Ltd, Worcestershire, UK) at 25°C. Each measuremnt was performed in triplicate.

The morphological study of the prepared Dox/P45 nanoparticles was carried out with TEM (JEM-1010, JEOL, Japan). In brief, the nanoparticle was dissolved in PBS and dipped on a carbon coated copper grid. The grid was air-dried at RT and then imaged with a TEM equipped at 80 kV acceleration voltage.

### Drug release studies

The *in vitro* release kinetics of Dox from the Dox/P45 nanoparticle was evaluated using a dialysis method. Briefly, 2 mL aqueous Dox/P45 nanoparticles were contained in a dialysis bag (MWCO, 5500 Da). The dialysis bag was placed in a beaker containing 30 mL PBS buffer of desired pH with continuous stirring at 37°C. A volume of 2ml PBS was periodically withdrawn and the same volume of fresh PBS was complemented. The released DOX was measured by UV-vis spectrophotometry at 485 nm. The cumulative release of Dox within 6 h was plotted over time.

### Cellular uptake and flow cytometric analysis

A549 cells, MCF-7 breast cancer cells and Human Embryonic Kidney 293 (HEK) normal cells were used to assess cellular uptake of Dox/P45 nanoparticle by fluorescence activated cell analysis (FACS) (BD Bioscience Mountain View, CA, USA). Briefly, Dox/P45, Dox/P41 nanoparticles or free Dox added to the cells at an equal DOX concentration of 20 μg/mL and incubated for 2 h at 37°C. The cells were rinsed twice with PBS and then trypsinized and resuspended in 500 μL of PBS. About 1×10^4^ cells were used for each FACS assay and the cellular uptake was assessed by the red fluorescence intensity. PBS-treated cells were used as a control.

### Confocal laser scanning microscopy (CLSM)

Cellular uptake of Dox/P45 nanoparticles was visualized with confocal microscopy. A549 cells, MCF-7 cells or HEK cells were seeded in a petri-dish and grown for 24 h at 37°C followed by an incubation with Dox/P45 nanoparticles, Dox/P41 nanoparticles or free Dox for 2 h at 37°C. Subsequently, the medium was discarded and the cells were washed with PBS buffer for 3 times. The

$$\text{DL}\left(\%\right)=\left({\text{W}}_{\text{Dox}}/{\text{W}}_{\text{Dox}/\text{P}45}\right)\times 100$$

cellular nuclei were stained with Hoechst 33342 for 10 min at room temperature. The fluorescent images of cells were observed using a laser scanning confocal microscope (CLSM, Leica TCP SP5). DOX was excited at 485 nm with the emission at 595 nm.

### CCK-8 assay

Cytotoxicity of Dox/P45 nanoparticles was evaluated by CCK-8 assay in HEK293 cells, A549 cells and MCF-7 cells. Briefly, the cells were seeded in a 96-well plate at a density of 1 ×10^4^ cells per well in RPMI 1640 with 10% fetal bovine serum (FBS) and 1% antibiotic-antimycotic and then incubated for 24h at 37°C with 5% CO2. Free Dox, Dox/P41 or Dox/P45 nanoparticles were dissolved in fresh medium and then added to each well. The plate was incubated for 2 h and then washed twice with PBS. After 48 h of incubation, a 10-uL of CCK-8 was added to each well and the cells were further incubated for 4 h and the absorbance of each well was measured using a Microplate Reader (Model 680, BIO-RAD, USA) at the wavelength of 450 nm. Cell viability was calculated according to the following equation:

Cell viability(%)=[(A−C)/(B−C)]×100

Where, A was the absorbance of the cells incubated with free Dox, Dox/P41 or Dox/P45 nanoparticles. B was the absorbance of the cells without DOX or nanoparticles addtion, and C was the absorbance of the blank medium.

### Statistical analysis

All data in tables and figures are expressed as the means ± standard deviations (SDs). Statistical analysis was performed using Student's t test. All statistical tests were two-sided, and P < 0.05 were considered statistical significant.

## SUPPLEMENTARY FIGURES


