# Supplementary material for: Doxorubicin-triggered self-assembly of native amphiphilic peptides into spherical nanoparticles
Source: Oncotarget. 2016 Aug 11;7(36):58445–58. doi: 10.18632/oncotarget.11213 (PMC5295442; doi:10.18632/oncotarget.11213)
Supplement: Supplementary file 1 [file oncotarget-07-58445-s001.pdf]

Doxorubicin-triggered self-assembly of native amphiphilic peptides into spherical nanoparticles

SUPPLEMENTARY FIGURES

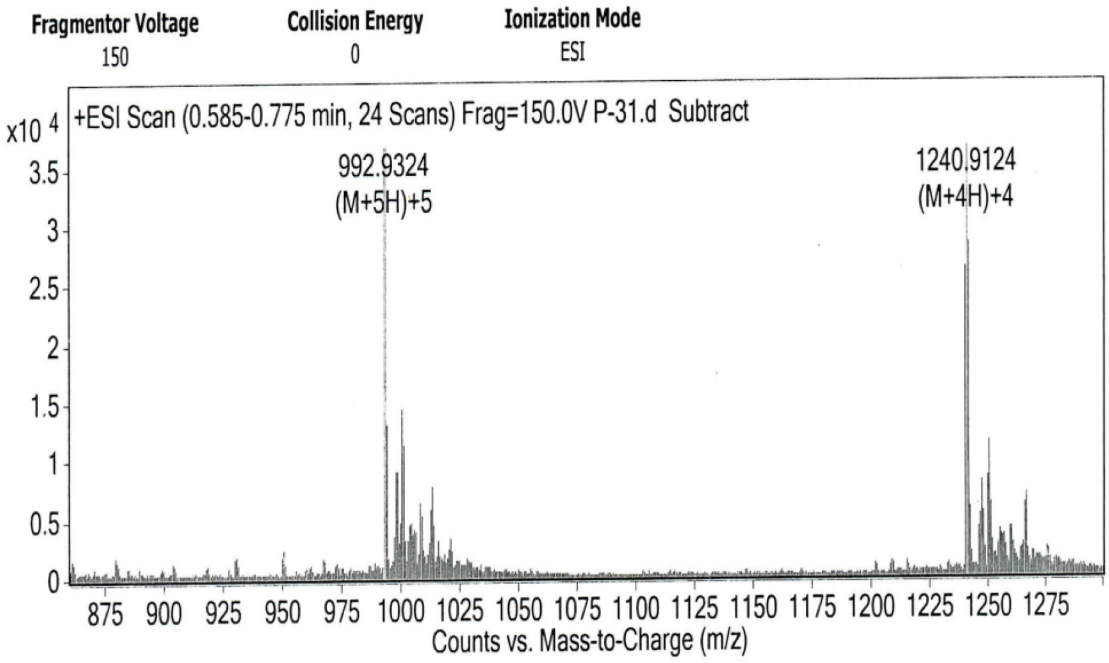

Supplementary Figure S1: The molecular weight of P45 peptide by mass spectrum matched with the theoretical value.

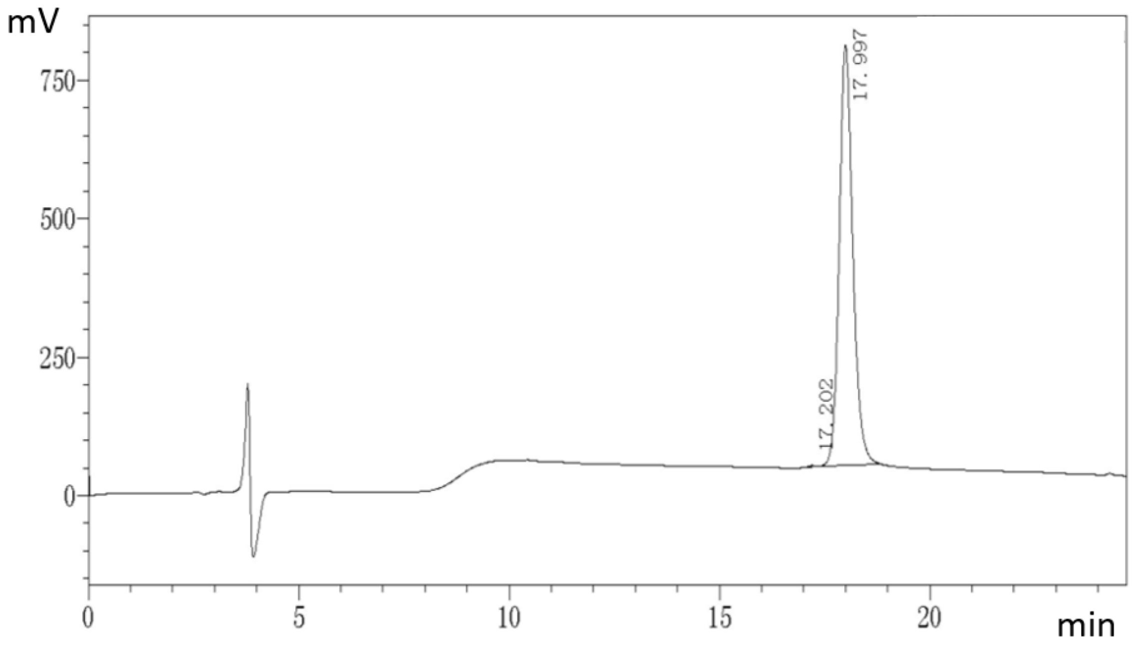

Number	Time	Acreage	Height	Concentration	Acreage(%)
1	17.202	22358	2520	0.131	0.131
2	17.997	17064463	758584	99.869	99.869
Total		17086821	761104		100.000

Supplementary Figure S2: The final product was obtained with high purity after purification by HPLC. Detector A Ch1 214nm

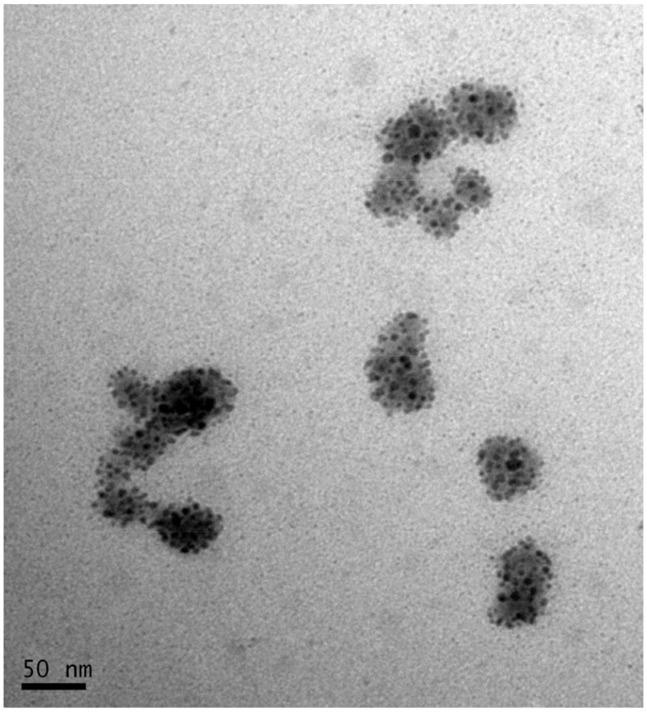

Supplementary Figure S3: Transmission electron microscopy (TEM) image of Dox/P41 nanoparticles. P41 was a truncated P45 without the RGDS motif at the N-terminal.
